# Neuroprotective Effects of Furanoditerpenes from *Spongia (Spongia) Tubulifera* through Cyclophilin D Modulation against Ischemia/Reperfusion Injury in BV2 Microglial Cells

**DOI:** 10.1021/acschemneuro.5c00949

**Published:** 2026-03-11

**Authors:** Noelia Castedo, Amparo Alfonso, Rebeca Alvariño, Dawrin Pech-Puch, Lucía Ageitos, Jaime Rodríguez, Mercedes R. Vieytes, Carlos Jiménez, Luis M. Botana

**Affiliations:** † Departamento de Farmacología, Facultad de Veterinaria, IDIS, 16780 Universidad de Santiago de Compostela, Lugo 27002, España; ‡ Departamento de Fisiología, Facultad de Veterinaria, IDIS, Universidad de Santiago de Compostela, Lugo 27002, España; § Centro Interdisciplinar de Química e Bioloxía (CICA) e Departamento de Química, Facultade de Ciencias, 16737 Universidade da Coruña, 15071 A Coruña, España; ∥ Escuela Nacional de Estudios Superiores Unidad Mérida (ENES Mérida), 7180 Universidad Nacional Autónoma de México (UNAM), Carretera Mérida-Tetiz km 4.5, Tablaje Catastral No. 6998, Municipio de Ucú, Ucú CP, Mexico 97357, Mexico

**Keywords:** cyclophilin D, furanoditerpenes, inflammation, ischemia, microglia

## Abstract

Ischemia induces oxidative stress and mitochondrial dysfunction in microglia, contributing to neuro-inflammation and neuronal damage. Five furanoditerpenes **1**–**5**, isolated from the marine sponge *Spongia (Spongia) tubulifera*, have previously shown neuroprotective effects related to their capacity to bind cyclophilin D (CypD), a protein involved in ischemia. In this study, the ability of compounds **1**–**5** to alleviate ischemic damage was evaluated on BV2 microglial cells. First, cells were incubated under oxygen deprivation for 6 h, and the five compounds were able to improve cell viability at micromolar concentrations (0.001–1 μM). Then, hypoxia was combined with the inflammatory stimulus lipopolysaccharide and with glucose deprivation, and *Spongia tubulifera* metabolites maintained their protective effects. When oxygen and glucose deprivation was followed by 6 h of reperfusion, compounds **1**–**5** also mitigated the damage produced on microglia. Moreover, these furanoditerpenes reduced reactive oxygen species overproduction and restored mitochondrial membrane potential, key factors in ischemic damage. This effect was mediated by the regulation of CypD since compounds **2**, **4**, and **5** reduced its expression under ischemia conditions. Finally, *trans*-well coculture experiments were performed between microglial and SH-SY5Y neuronal cells. In this assay, compounds **2**, **4**, and **5** protected neuronal cells from microglial-induced neurotoxicity under ischemia/reperfusion conditions. These findings suggest that *S. tubulifera* metabolites display mitochondrial-mediated antioxidant and cytoprotective effects under ischemic conditions through CypD modulation. Given the limitations of current Cyps inhibitors like cyclosporin A, compounds **1**–**5** are promising therapeutic candidates for ischemia-related diseases, such as stroke.

## Graphical abstract

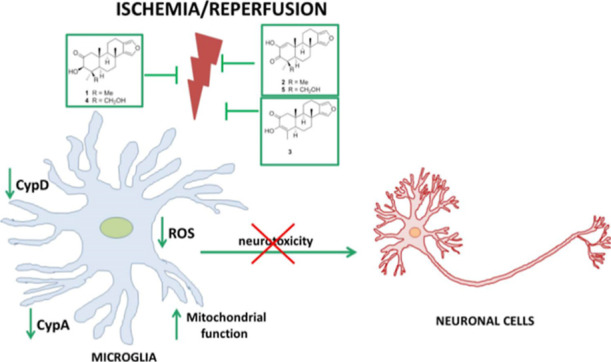

## 1 Introduction

Sponge (*Spongia tubulifera)* is a source of several natural compounds of different chemical nature.^1,2^ Five compounds extracted from *S. tubulifera* with furanoditerpene structure have previously shown neuroprotective properties through a direct interaction with cyclophilin D (CypD).^3^ This protein is the only mitochondrial isoform from a protein family, cyclophilins (Cyps), with peptidyl-prolyl isomerase activity and high affinity for cyclosporine A (CsA).^4^ Metabolites from *S. tubulifera* have also shown affinity for CypA, a cytosolic member of Cyps family associated with inflammation and immune response.^3,5^ Cyps have been related to ischemia processes since CypD stabilizes the mitochondrial transition permeability pore (mPTP) opening during ischemia, a channel that causes a massive outflow of ions leading to mitochondrial swelling and cell death.^4^ In addition, CypA has been implicated in proinflammatory signaling during ischemia injury.^6^ Thus, modulation of Cyps with compounds from *S. tubulifera* could represent a promising alternative to CsA, the only Cyp-targeting drug currently approved for clinical use, which is associated with many side effects.^7^

Ischemia results from a deprivation of blood and/or oxygen to the organ, which leads to cellular hypoxia followed by reperfusion when blood flow and oxygen are restored.^8^ The brain is more sensitive than other organs to changes in blood and oxygen levels since it needs a constant supply of nutrients and oxygen to function properly.^9^ For this reason, a prolonged state of hypoxia initiates multiple damaging mechanisms and triggers many pathophysiological changes, such as energy failure, oxidative stress, endothelial damage and neuronal death.^10^ Indeed, after brain hypoxia, damaged neurons release neuromodulators and increase reactive oxygen species (ROS) content, activating microglial cells.^11^ Microglia are resident macrophage-like immune cells of the central nervous system (CNS) that represent the first line of defense against damage and play a pivotal role in the regulation of the inflammatory response in the brain.^12^ These cells undergo phenotype changes that range from a homeostatic state under physiological conditions to a reactive phenotype when immunological homeostasis is disrupted.^13^ Nevertheless, microglia exhibit great heterogeneity under hypoxia. After ischemia, there is a differential shift from the surveillant phenotype to the reactive phenotype, which exacerbates ischemic injury.^11^ In this state, microglial activation leads to an impaired mitochondrial function that results in an increased production of ROS and changes in the mitochondrial membrane potential (ΔΨm) and ATP production.^14^ After hypoxia, there is a rapidly restoration of blood flow that may aggravate cerebral damage by increasing oxidative stress, generating calcium overload, enhancing mitochondrial dysfunction and activating mitochondrial-dependent apoptosis.^15^ Therefore, inhibition of microglial activation under hypoxia/reperfusion conditions could be an essential strategy to facilitate better recovery after brain ischemia.

In this work, five compounds **1**–**5** (Figure 1a) isolated from the sponge *S. tubulifera* were tested in BV2 microglial cells to determine their protective activity under hypoxia and hypoxia/reperfusion injury.

**Figure 1:**
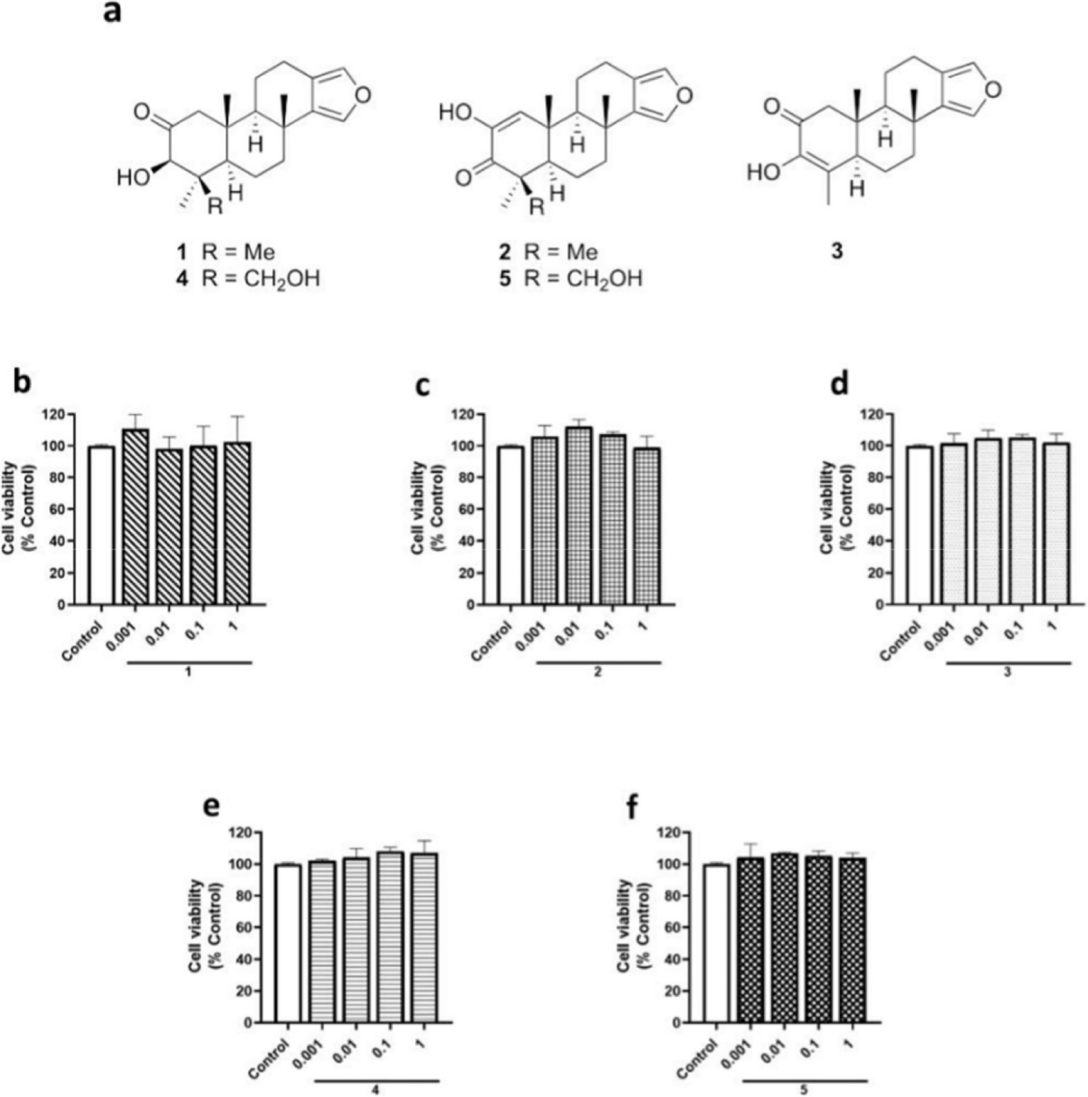
Chemical structures and effect of compounds **1**–**5** on the cell viability of BV2 microglial cells. Compounds were added to murine microglial cells for 24 h under normoxia conditions and cell viability was assessed. (A) Chemical structure of compounds **1**–**5**. Compound **1** (b), compound **2** (c), compound **3** (d), compound **4** (e), and compound **5** (f) effects on cell viability. Data are mean ± SEM of three independent experiments performed in triplicate. Data are expressed as percentage of control cells.

## 2 Experimental Section

### 2.1 Chemicals and Solutions

Tetramethylrhodamine methyl ester (TMRM) and 5-(and 6)-carboxy-2′,7′-dichlorodihydrofluorescein diacetate (carboxy-H_2_DCFDA) were of reagent grade and purchased from Thermo Fisher Scientific (Madrid, Spain). CsA with a purity ≥98.5% was obtained from Abcam (Cambridge, UK). Lipopolysaccharide (LPS) was obtained from *Escherichia coli* O111:B4, 3-(4,5-dimethylthiazol-2-yl)-2,5-diphenyl tetrazolium bromide (MTT) dye, and the rest of the chemicals and reagents were obtained from Sigma-Aldrich (Madrid, Spain). The composition of the saline solution (Locke’s Buffer) used for the viability and ΔΨm assays was (in mM): 154 NaCl, 5.6 KCl, 1.3 CaCl_2_, 1 MgCl_2_, 3.6 NaHCO_3_, 5.6 glucose, and 10 HEPES. The composition of the lysis buffer used to obtain the cytosolic fraction was (in mM): 20 Tris–HCl (pH 7.4), 10 NaCl, and 3 MgCl_2_, containing phosphatase and protease inhibitor cocktails. The phosphate-buffered saline (PBS) solution was composed by 137 mM NaCl, 8.2 mM Na_2_HPO_4_, 1.5 mM KH_2_PO_4_, and 3.2 mM KCl.

### 2.2 Extraction and Isolation of Compounds **1–5**

Compounds **1–5** were obtained from the sponge *S. tubulifera* collected from the Mexican Caribbean coast. All compounds are >95% pure by HPLC and NMR, as previously described.^2^

### 2.3 Cell Culture

Murine microglial BV2 cell line, purchased from Interlab Cell Line Collection (ICLC) number ATL0300 (Genova, Italy), were maintained in RPMI 1640 medium with l-glutamine and phenol red at 37 °C in a humidified atmosphere of 5% CO_2_ and 95% air. Medium was supplemented with 10% fetal bovine serum (FBS), penicillin (100 U/mL), and streptomycin (100 μg/mL). Cells were dissociated twice a week with 0.05% trypsin/EDTA.

Human neuroblastoma SH-SY5Y cell line was purchased from American Type Culture Collection (ATCC), number CRL2266. Cells were maintained in Dulbecco’s modified Eagle’s Medium: Nutrient Mix F- 12 (DMEM/F- 12) enriched with 10% FBS, 1% glutamax, penicillin (100 U/mL), and streptomycin (100 μg/mL). Cells were dissociated weekly using 0.05% trypsin/EDTA and maintained at 37 °C in a humidified atmosphere of 5% CO_2_ and 95% air.

### 2.4 Ischemia Model

Cells were seeded in 384-well plates at a density of 2 × 10^4^ cells per well. After 24 h, cells were treated with compounds at nontoxic concentrations (0.001–1 μM) for 6, 12, or 24 h under oxygen restriction (94% N_2_, 5% CO_2_, 1% O_2_) (hypoxia) and/or glucose deprivation (OGD) in a hypoxia incubator chamber (StemCell Technologies, Canada). To establish the hypoxia/reperfusion model, plates incubated 6 h under OGD were then reperfused with oxygen and medium was changed to a glucose-containing medium (OGD/REP). A control plate per each time point was seeded in normal RPMI medium and maintained at 37 °C, 5% CO_2_, and 95% air.

### 2.5 Cell Viability Assay

The ability of the compounds to protect cells from the hypoxia injury was determined via MTT dye.^16^ After the previously described treatment, cells were washed three times with saline buffer (Locke’s buffer). Then, 100 μL of MTT (500 μg/mL), dissolved in the saline solution, was added to each well followed by an incubation of 1 h at 37 °C. Afterward, 100 μL of 5% sodium dodecyl sulfate (SDS) was added. Formation of formazan crystals was measured at 595 nm with a plate reader. Saponin at 1 mg/mL was used as a death control. Experiments were performed in triplicate at least three independent times.

### 2.6 ROS Assay

Intracellular ROS levels were determined with the fluorescent dye carboxy-H_2_DCFDA, as previously described.^17^ After treatment, explained before, cells were washed twice with serum-free RPMI medium and 100 μL of 20 μM carboxy-H_2_DCFDA was added to each well. After 1 h of incubation at 37 °C and 300 rpm, the dye was removed and 100 μL of PBS was added. Cells were incubated for 30 min at 37 °C and 300 rpm, and ROS levels were measured at 495 nm excitation and 527 nm emission with a spectrophotometer plate reader. All measurements were performed in triplicate at least three independent times.

### 2.7 Mitochondrial Membrane Potential Assay

To analyze the effect of compounds in ΔΨm, fluorescent dye TMRM was used. Briefly, after treatment, cells were washed twice with Locke’s solution, and 1 μM TMRM was added to each well. After 30 min of incubation at 37 °C and 300 rpm, cells were lysed with DMSO at 50%. The ΔΨm was measured in a plate reader at 535 nm excitation and 590 nm emission. All measurements were performed in triplicate at least three independent times.

### 2.8 Microglia and Neuron Coculture System

Neuroblastoma SH-SY5Y cells were grown in a 24-well plate, while BV2 microglial cells were grown in culture inserts (pore size 0.4 μm), and placed above the plate.^18^ Inserts containing microglia were treated with compounds (0.1 μM) or CsA (1 μM) 1 h before OGD/REP incubation for 6 h under the OGD followed by 6 h under the REP. Then, cellular inserts were removed and the MTT assay was performed in SH-SY5Y cells, as described above.

### 2.9 Protein Extraction

A total of 1 × 10^6^ cells per well were seeded in 12-well plates and incubated under OGD/REP as previously described. Cells were washed twice with ice-cold PBS, and 100 μL of lysis buffer was added to each well to obtain the cytosolic protein fraction. Then, cells were incubated on ice for 15 min, and 5 μL of Triton X-100 was added. Cells were centrifuged at 3000 rpm and 4 °C for 10 min. The supernatant obtained was collected as the cytosolic fraction and quantified through a Direct Detect system (Merck, Germany).

### 2.10 Western Blot Analysis

Western blotting of the cytosolic content was performed after protein extraction. Briefly, 10 μg of cytosolic protein was resolved on a 4–20% polyacrylamide gel and proteins were transferred onto PVDF membranes via the Trans-Blot semidry transfer system. Protein weight was determined with the molecular-weight marker Precision Plus Protein Standard Kaleidoscope. Membranes were blocked with 0.5% BSA and incubated with antibodies using the SNAP i.d. system (Merck). CypA was detected with anti-PPIA primary antibody (1:1000, Elabscience), and CypD was distinguished with anticyclophilin F primary antibody (1:1000, Abcam). The specificity of Cyps antibodies was previously tested.^3^ The detection of specific protein bands was performed by using Supersignal West Pico or Supersignal West Femto. The intensity of the protein bands was normalized using anti-Actin (1:2000, Sigma-Aldrich). Protein bands were densitometrically analyzed by using Diversity GeneSnap (Syngene). All measurements were performed in duplicate in three independent experiments.

### 2.11 Statistical Analyses

Data are presented as mean ± SEM. Statistical differences were evaluated by one way ANOVA with Dunnett’s or Tuckey’s post hoc test using GraphPad Prism v.10 software. Statistical significance was considered at *p* < 0.05.

## 3 Results

The effect of five compounds (**1**–**5**) isolated from *S. tubulifera* under oxidative stress was previously studied in neurons,^3^ but there are no data available regarding the effect of these metabolites under hypoxia, OGD, or OGD/REP injury. In this context, the aim of this work was to determine the effect of compounds **1**–**5** under these conditions in BV2 microglial cells. In all the assays, the immunosuppressant drug CsA was used as the positive control of anti-inflammatory effects through Cyps inhibition.

First, the cytotoxic effects of compounds **1**–**5** (Figure 1a) on BV2 microglial cells were evaluated. Compounds were added at concentrations that ranged from 0.001 to 1 μM to microglial cells for 24 h under normal oxygen conditions or normoxia. None of the concentrations of compounds **1**–**5** affected BV2 viability after 24 h of treatment (Figure 1b–f).

Therefore, microglia were incubated under 6, 12, or 24 h of hypoxia with metabolites from *S. tubulifera* at 0.001, 0.01, 0.1, and 1 μM. Cell viability was reduced by 25% after 6 h, by 47% after 12 h, and by 61% after 24 h of hypoxia compared to the normoxia control, and in these conditions, the control of CsA showed no effect (Figure 2). After 6 h of hypoxia incubation, when microglia were treated with compound **1**, 0.1 and 1 μM concentrations significantly improved the effect of hypoxia by 11 and 21%, respectively (Figure 2a). When microglia were exposed to 12 h of hypoxia, compound **1** also counteracted the effect of hypoxia at 0.001, 0.1, and 1 μM (Figure 2b). Indeed, compound **1** also reduced the cytotoxicity of hypoxia at all the used concentrations after 24 h (Figure 2c). On the other hand, compound **2** displayed a significant improvement of the cytotoxic effects under 6 h of hypoxia at all the doses assayed, being higher at the lowest concentration (recovery of 36% of hypoxia control cells) (Figure 2d). After 12 h of hypoxia, compound **2** mitigated hypoxia impact over cell viability reaching control levels at all the compound concentrations (Figure 2e). In addition, the effect of 24 h of hypoxia was also attenuated when microglia were treated with compound **2** at 0.1 and 1 μM (Figure 2f). When microglia were incubated with compound **3** under 6 or 12 h of hypoxia, only 0.001 and 0.01 μM inhibited the reduction of cell viability, reaching control levels (Figure 2g,h). In contrast, after 24 h of hypoxia, only 0.01 and 0.1 μM concentrations mitigated the impact of hypoxia with levels of 63.6 ± 4.0% and 54.3 ± 2.6% of control cells, respectively (Figure 2i). Compound **4** prevented the hypoxia-induced reduction in microglial viability after 6 h with a dose-dependent response (Figure 2j). In addition, after 12 h of hypoxia, compound **4** at 0.01 and 0.1 μM showed a significant effect (Figure 2k). However, compound **4** exhibited a significant effect mitigating the reduction of cell viability after 24 h of hypoxia at all the concentrations used (Figure 2l). Regarding compound **5**, incubation under 6 or 12 h in hypoxia conditions improved BV2 cells viability reduction at all the concentrations (Figure 2m,n). In fact, treatment with compound **5** also reduced the impact of 24 h of hypoxia at all the concentrations, reaching levels of 76% of control cells at 0.1 μM (Figure 2o). In summary, BV2 microglial viability was markedly time-dependently reduced after hypoxia incubation. In these conditions, treatment with compounds **1**–**5** attenuated the effect of hypoxia with a higher impact after 6 h, being compounds **2**, **3**, and **5** the most effective ones.

**Figure 2:**
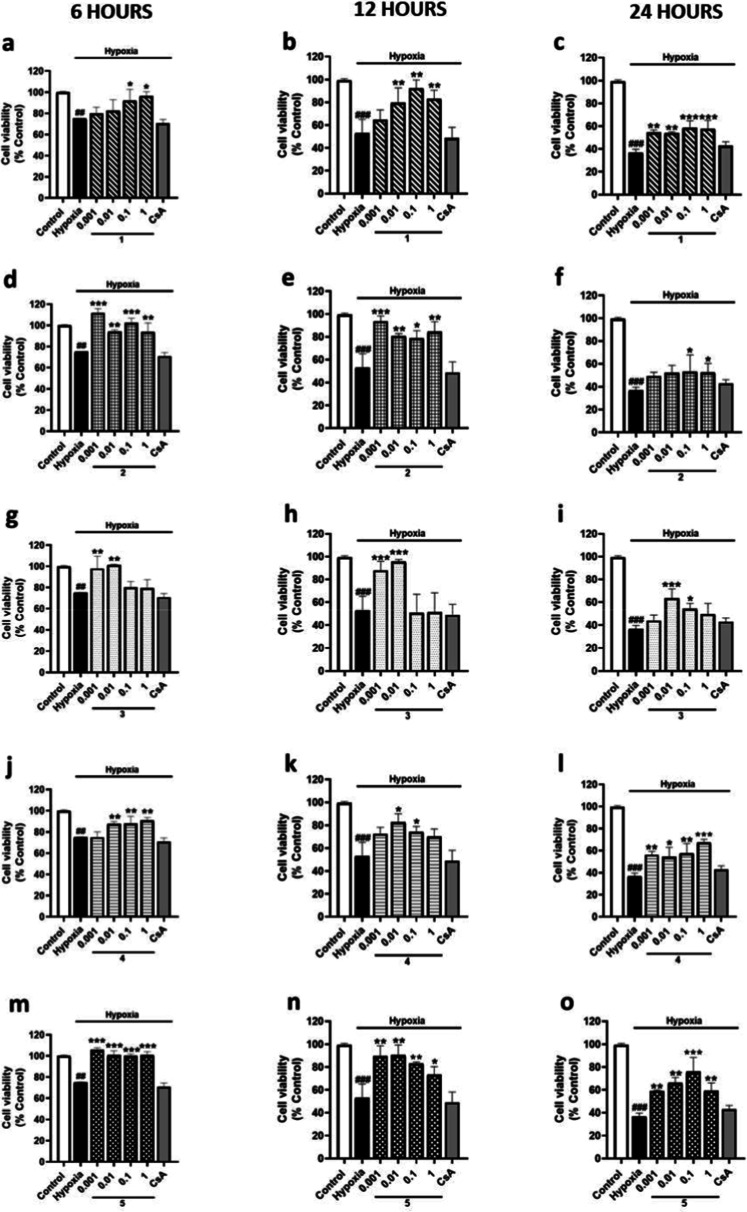
Effect of compounds **1**–**5** on the cell viability of BV2 microglial cells under 6, 12, or 24 h of hypoxia. CsA (1 μM) was used as the positive control of Cyps route. Results of compound **1** under 6 (a), 12 (b), or 24 h of hypoxia (c). Results of compound **2** under 6 (d), 12 (e), or 24 h of hypoxia (f). Results of compound **3** under 6 (g), 12 (h), or 24 h of hypoxia (i). Results of compound **4** under 6 (j), 12 (k), or 24 h of hypoxia (l). Results of compound **5** under 6 (m), 12 (n), or 24 h of hypoxia (o). Data are mean ± SEM of three independent experiments performed in triplicate. Data are expressed as percentage of control cells in normoxia conditions. Statistical differences were determined by one-way ANOVA and Dunnett’s tests. ##*p* < 0.01 and ###*p* < 0.001 compared to control cells. *p < 0.05, **p < 0.01, and ***p < 0.001 compared to hypoxia cells.

Given that most of the metabolites from *S. tubulifera* protected BV2 cells from the hypoxia-associated damage, the following step was to determine their potential when hypoxia is combined with the inflammatory stimulus LPS. Therefore, microglia were incubated with compounds **1**–**5** at 0.001, 0.01, 0.1, and 1 μM in combination with 500 ng/mL LPS under 6, 12, or 24 h of hypoxia. When microglial viability under 6, 12, or 24 h hypoxia was analyzed, no significant effect of LPS combined with hypoxia was observed compared to hypoxia cells, while the control of CsA showed no effect (Figure 3). In these conditions, after 6 h, compound **1** significantly improved the reduction of microglial viability reaching control levels at all the concentrations (Figure 3a). In contrast, the effect of compound **1** was lower after 12 or 24 h and higher concentrations were needed (Figure 3b,c). Regarding compound **2**, all the used concentrations combined with LPS also inhibited the impact of 6 or 12 h of hypoxia, reaching control levels (Figure 3d,e). Nevertheless, higher compound **2** concentrations were required to counteract the effect of 24 h of hypoxia (Figure 3f). When microglia were treated with compound **3** under LPS-hypoxia incubation, all the concentrations showed a significant effect over microglial viability reduction after 6 or 12 h (Figure 3g,h), while no effect was observed under 24 h (Figure 3i). On the other hand, compound **4** reduced the cytotoxic effects of 6 h of LPS and hypoxia at all the doses, with higher effect at lower concentrations (Figure 3j), as well as after 12 h of hypoxia (Figure 3k). However, the efficiency of compound **4** under 24 h of hypoxia was reduced (Figure 3l). Finally, compound **5** significantly ameliorated cell survival reduction at all the tested concentrations after 6, 12, or 24 h with levels among control cells (Figure 3m,n,o). In that way, when microglia were exposed to hypoxia, LPS did not enhance the reduction in microglial viability after 6, 12, or 24 h. Moreover, under these conditions, compounds **1**–**5** were able to reduce the cytotoxic effects of hypoxia combination with LPS, with a higher effect under 6 h. Since compounds **1**–**5** showed a higher effect after 6 h of hypoxia, the following experiments were conducted at this incubation time. In addition, 0.01, 0.1, and 1 μM doses were chosen since they were the most effective concentrations.

**Figure 3:**
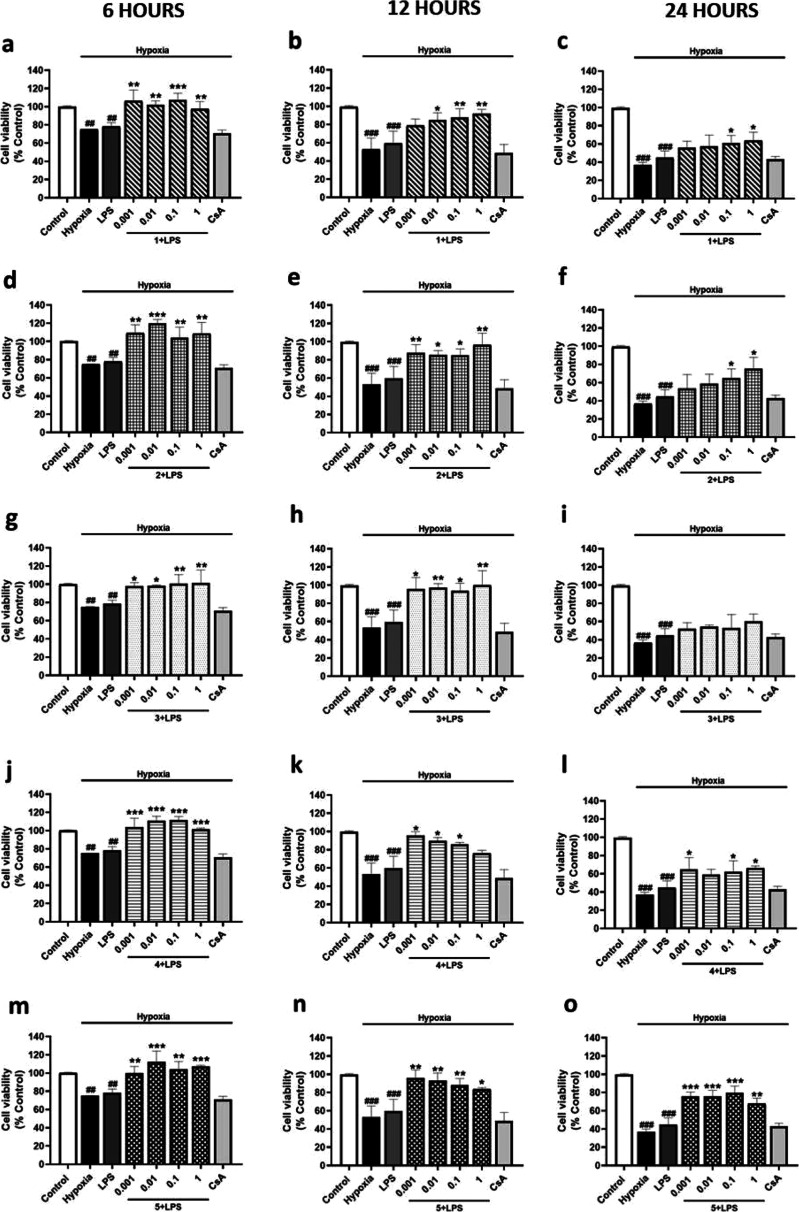
Cell viability of BV2 cells treated with compounds **1**–**5** and LPS under 6, 12, or 24 h of hypoxia. Compounds were added together with LPS (500 ng/mL) to murine microglial cells under hypoxia. CsA (1 μM) was used as the positive control of Cyps route. Results of compound **1** under 6 (a), 12 (b), or 24 h of hypoxia (c). Results of compound **2** under 6 (d), 12 (e), or 24 h of hypoxia (f). Results of compound **3** under 6 (g), 12 (h), or 24 h of hypoxia (i). Results of compound **4** under 6 (j), 12 (k), or 24 h of hypoxia (l). Results of compound **5** under 6 (m), 12 (n), or 24 h of hypoxia (o). Data are mean ± SEM of three independent experiments performed in triplicate. Data are expressed as percentage of control cells in normoxia. Statistical differences were determined by one-way ANOVA and Dunnett’s tests. ##*p* < 0.01 and ###*p* < 0.001 compared to control cells. *p < 0.05, **p < 0.01, and ***p < 0.001 compared to LPS-treated cells under hypoxia.

The following step was to determine the combined effect of oxygen and glucose deprivation (OGD) over the viability of microglia as it happens under physiological conditions. Therefore, microglia were exposed to 6 h of oxygen-glucose deprivation (OGD) and treated with metabolites from *S. tubulifera* at 0.01, 0.1, and 1 μM. After 6 h of OGD, cell viability was significantly reduced by 31% compared to the normoxia control cells, and CsA reduced the cytotoxic effects of OGD by 12% (Figure 4). In these conditions, when microglia were treated with compound **1**, a significant dose-dependent effect on cell viability was observed, with levels of 82.7 ± 4.1% at 1 μM (Figure 4a). Regarding compound **2**, all the assayed concentrations showed a protective effect, with levels among 88.6% at 0.01 μM and 83.3% at 1 μM (Figure 4b). Treatment with compound **3** counteracted the effect of OGD at all the assayed concentrations, reducing cytotoxicity by 17% at 1 μM (Figure 4c). Compounds **4** and **5** also showed protective effects against OGD at all the dosses assayed in a dose-dependent way, reducing the cytotoxic effects by 20% at the highest concentration (Figure 4d,e).

**Figure 4:**
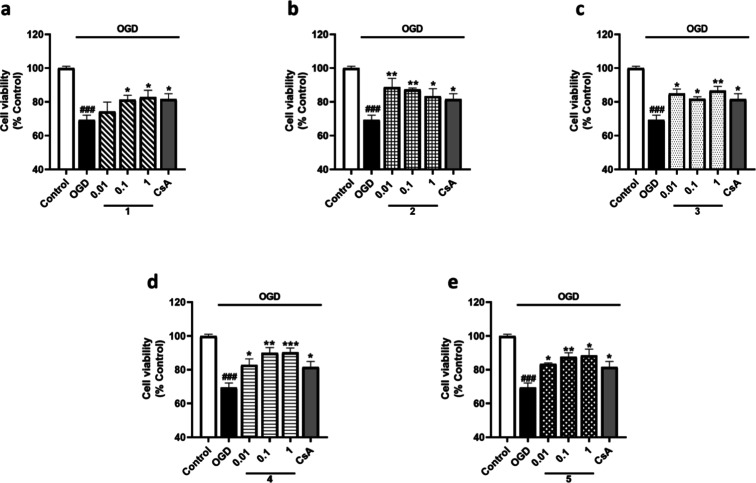
Evaluation of the viability of microglial cells treated with *S. tubulifera* metabolites after 6 h of OGD incubation. Compounds were added to murine microglial cells for 6 h under OGD. CsA (1 μM) was used as the positive control of Cyps route. Results from (a) compound **1**, (b) compound **2**, (c) compound **3**, (d) compound **4**, and (e) compound **5**. Data are mean ± SEM of three independent experiments performed in triplicate. Data are expressed as percentage of control cells in normoxia. Statistical differences were determined by one-way ANOVA and Dunnett’s tests. ###*p* < 0.001 compared to control cells. *p < 0.05, **p < 0.01, and ***p < 0.001 compared to OGD control cells.

In view of compounds **1**–**5** being able to reduce the effect of OGD, cells were incubated under the OGD/REP conditions to study the potential effect of these compounds in reperfusion. Therefore, microglia were exposed to 6 h of OGD followed by 6 h under REP together with metabolites from *S. tubulifera* at 0.01, 0.1, and 1 μM. After OGD/REP incubation, cell viability was significantly reduced by 40% compared to the control cells and the control of CsA counteracted this response by 10% (Figure 5). Under these conditions, compound **1** mitigated the effect of the OGD/REP by 11% at the highest dose (Figure 5a). Compound **2** significantly reduced the cytotoxic effects of OGD/REP in a dose-dependent way with levels of 77.3 ± 2.9% at 1 μM (Figure 5b). In addition, compound **3** also showed a significant effect at all the assayed concentrations, reducing the effect of the OGD/REP by 20% at 0.1 μM (Figure 5c). When cells were treated with compounds **4** and **5**, a significant effect of 15% over the reduction of cell viability was observed at 0.1 μM (Figure 5d,e). As the results showed, the viability of BV2 microglial cells under hypoxia or OGD/REP was reduced compared to control cells, and in these conditions, compounds **1**–**5** protected microglia.

**Figure 5:**
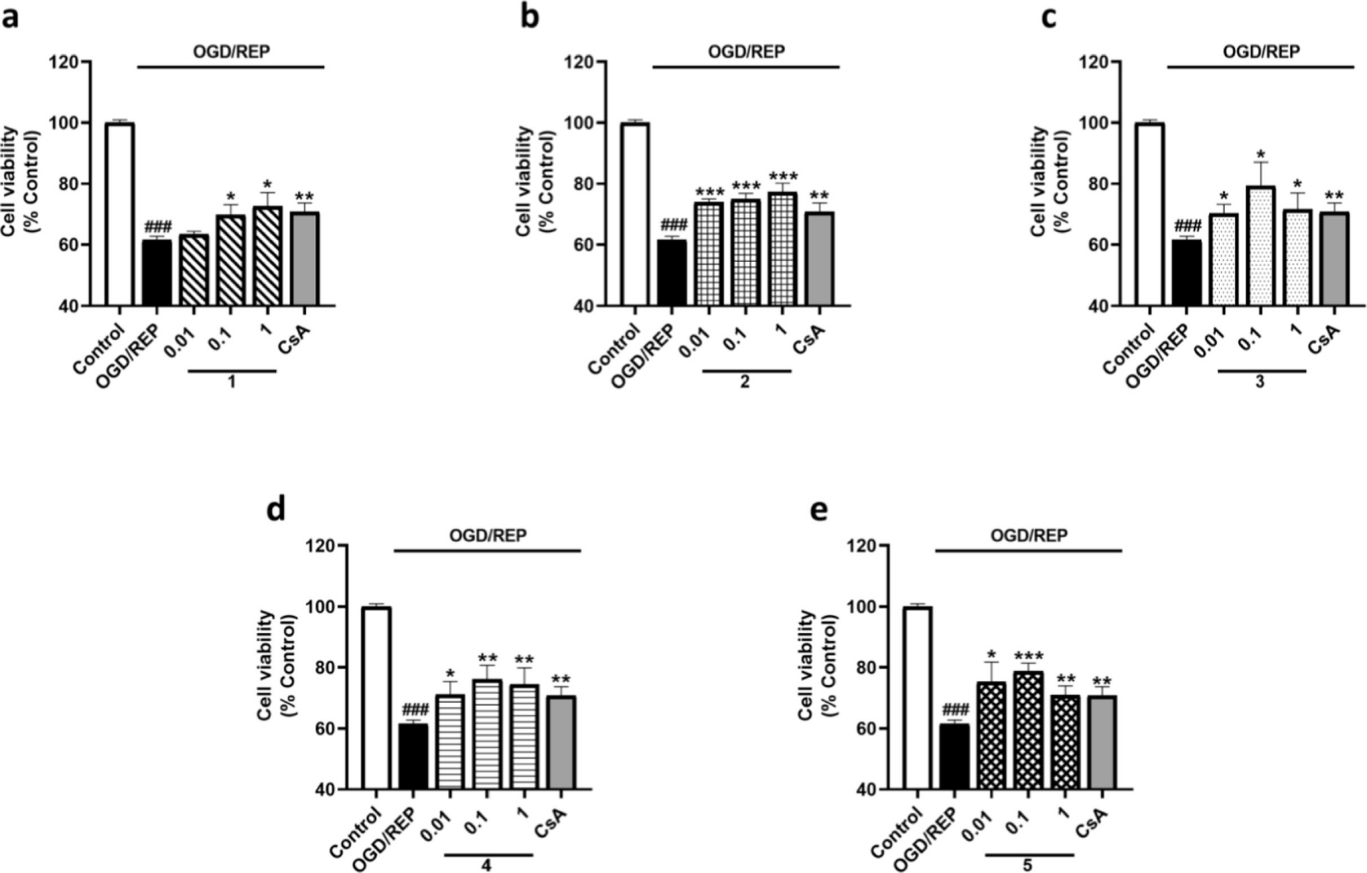
Effect of compounds **1**–**5** on BV2 cells viability after OGD/REP incubation. Compounds were added to murine microglial cells for 6 h under OGD followed by 6 h of REP. CsA (1 μM) was used as the positive control of Cyps route. Compound **1** (a), compound **2** (b), compound **3** (c), compound **4** (d), and compound **5** (e) results. Data are mean ± SEM of three independent experiments performed in triplicate. Data are expressed as percentage of control cells in normoxia. Statistical differences were determined by one-way ANOVA and Dunnett’s tests. ###*p* < 0.001 compared to control cells. *p < 0.05, **p < 0.01, and ***p < 0.001 compared to OGD/REP control cells.

Next, ROS intracellular levels after treatment with compounds under 6 h hypoxia or OGD/REP were evaluated. These results are shown as increments of ROS levels of control cells. ROS were significantly increased under hypoxia or OGD/REP compared to control cells and, in these conditions, the control of CsA reduced ROS levels significantly (Figure 6). When compounds were added alone, no effect over ROS levels was observed (data not shown), while under hypoxia or OGD/REP compounds mitigated ROS release (Figure 6). Regarding compounds **1** and **2**, treatment in combination with hypoxia decreased ROS levels by 76% at 0.01 μM and 72% at 1 μM, respectively (Figure 6a,b). Incubation with compound **3** attenuated the increase in ROS levels under hypoxia, reaching a 74% reduction at 0.01 μM (Figure 6c). Compounds **4** and **5** were also effective under hypoxia with a 60% and 40% decrease of ROS increment at the highest dose, respectively (Figure 6d,e). Given that the minimum concentration that showed significant effects in cell viability and ROS assays at all of the incubation times was 0.1 μM, this dose was chosen to conduct the following experiments under the OGD/REP conditions. As shown in Figure 6f, the five compounds reduced ROS to control levels, being compounds **4** and **5** the most effective, since ROS levels were mitigated even under control cells levels.

**Figure 6:**
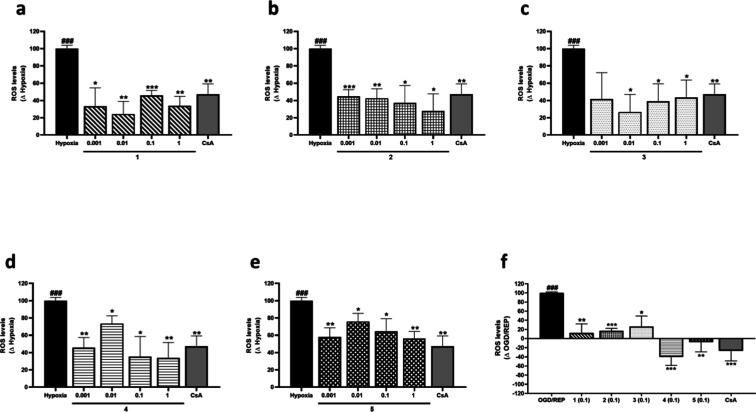
ROS intracellular levels after treatment with compounds **1**–**5** under hypoxia or OGD/REP. Compounds **1**–**5** were added to murine microglial cells for 6 h under hypoxia or OGD/REP conditions. CsA (1 μM) was used as the positive control of Cyps route. ROS levels after treatment with (a) compound **1**, (b) compound **2**, (c) compound **3**, (d) compound **4**, and (e) compound **5** under hypoxia. Results of (f) compounds **1**–**5** (0.1 μM) under OGD/REP. Data are mean ± SEM of three independent experiments performed in triplicate. Data are expressed as increment of normoxia control cells. Statistical differences were determined by one-way ANOVA and Dunnett’s tests. ###*p* < 0.001 compared to control cells. *p < 0.05, **p < 0.01, and ***p < 0.001 compared to hypoxia or OGD/REP control cells.

Next, mitochondrial function under hypoxia or OGD/REP incubation was determined by measuring the ΔΨm since mitochondria are one of the main producers of ROS (Figure 7). These results are shown as an increment or decrease of ΔΨm of control cells. As Figure 7 shows, 6 h of incubation under hypoxia induced the hyperpolarization of mitochondrial membrane, while after reperfusion mitochondrial membrane was depolarized. In both conditions, treatment with the control of CsA showed an 80% recovery of mitochondria from the hypoxia and OGD/REP injury. When compounds were added alone, no effect over ΔΨm was observed (data not shown), while under hypoxia or OGD/REP compounds counteracted mitochondrial membrane injury (Figure 7). Regarding compound **1**, when combined with hypoxia, ΔΨm was significantly decreased at all the tested concentrations, with an inhibition among 107 and 144% of hypoxia increment (Figure 7a). Compounds **2** and **3** were also able to decrease membrane hyperpolarization at the four tested concentrations (Figure 7b,c). In addition, compound **4** also reduced the hyperpolarization of the mitochondrial membrane when incubated in hypoxia at all the doses used, with an inhibition of 156% at 1 μM compared to the increment under hypoxia (Figure 7d). Finally, compound **5** also diminished the ΔΨm under control levels in a dose-dependent way (Figure 7e). In addition, the effect of compounds **1**–**5** on the depolarization observed under OGD/REP was analyzed. As previously explained, experiments in OGD/REP conditions were conducted with a compound concentration of 0.1 μM since it is the minimum concentration with a significant effect among the hypoxia incubation times. As Figure 7f shows, compounds **1**–**5** were able to recover mitochondria from the depolarization induced by OGD/REP. Compounds **2**, **4**, and **5** were the most effective, mitigating the reduction in the ΔΨm in 60–70% after treatment at 0.1 μM (Figure 7f). In summary, ROS levels were doubled by hypoxia or OGD/REP conditions, and compounds were able to reduce them, compounds **4** and **5** being the most effective after reperfusion. In addition, compounds **1**–**5** were also able to mitigate the impact of hypoxia or OGD/REP over the ΔΨm, displaying compounds **2**, **4**, and **5** the greatest effects. In view of previous results and since compound **3** showed discrepancies through hypoxia incubation times, compound **3** was not included in the following experiments.

**Figure 7:**
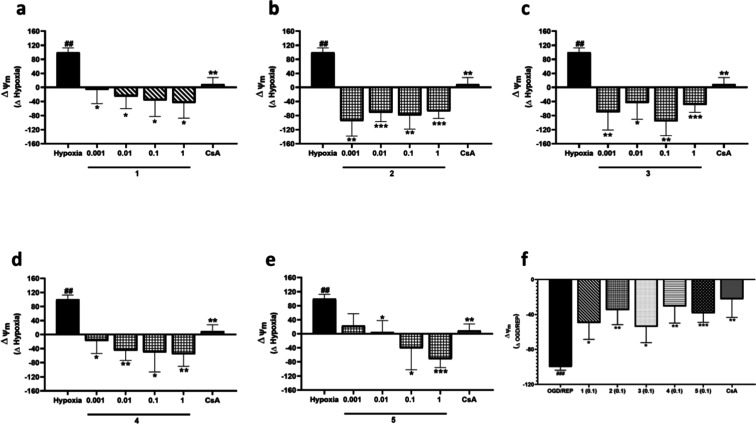
Effect of compounds **1**–**5** on ΔΨm of BV2 cells after hypoxia or OGD/REP. Compounds **1**–**5** were added to murine microglial cells for 6 h under hypoxia or OGD/REP conditions. CsA (1 μM) was used as the positive control of Cyps route. ΔΨm after addition of (a) compound **1**, (b) compound **2**, (c) compound **3**, (d) compound **4**, and (e) compound **5** under hypoxia. Results of (f) compounds **1**–**5** (0.1 μM) under OGD/REP. Data are mean ± SEM of three independent experiments performed in triplicate. Data are expressed as increment normoxia control cells. Statistical differences were determined by one-way ANOVA and Dunnett’s tests. ##*p* < 0.01 and ###*p* < 0.001 compared to normoxia control cells. *p < 0.05, **p < 0.01, and ***p < 0.001 compared to hypoxia or OGD/REP control cells.

Since CypD and CypA are the target of compounds **1**–**5**,^3^ the effect of metabolites from *S. tubulifera* on the expression of both proteins after ischemia/reperfusion injury was evaluated. Cytosolic levels of CypD and CypA were assessed after 6 h of OGD incubation followed by 6 h of REP together with compounds **1**, **2**, **4**, and **5** at 0.1 μM. Under OGD/REP incubation, CypD levels increased markedly compared to control cells (134.3 ± 11.7%), while the control of CsA reduced nearly half the expression of CypD (Figure 8a). In these conditions, treatment with compounds **2**, **4**, and **5** mitigated CypD expression, being compound **2** the most effective reducing CypD expression by 52%. Compounds **4** and **5** also decreased CypD expression up to 40–50% of the OGD/REP control cells (Figure 8a). Regarding CypA, when microglia were incubated in OGD/REP conditions, CypA levels were increased (116.9 ± 1.6%) and only compound **1** and CsA control decreased CypA intracellular expression by 30% (Figure 8b). BV2 cells exposed to compounds at 0.1 μM under normoxic conditions did not exhibit any significant changes in CypD or CypA expression, a similar effect observed with 1 μM CsA (Figure 8c,d). In summary, under the OGD/REP incubation, CypD and CypA levels were enhanced, and compounds **2**, **4**, and **5** were able to reduce CypD expression, while only compound **1** could decrease the intracellular levels of CypA. The control of CsA diminished the expression of both proteins. Given that CypD plays an important role in ischemia/reperfusion conditions and that compounds **2**, **4**, and **5**, which bind to this protein,^3^ showed a greater effect, the following experiment was conducted using these three compounds.

**Figure 8:**
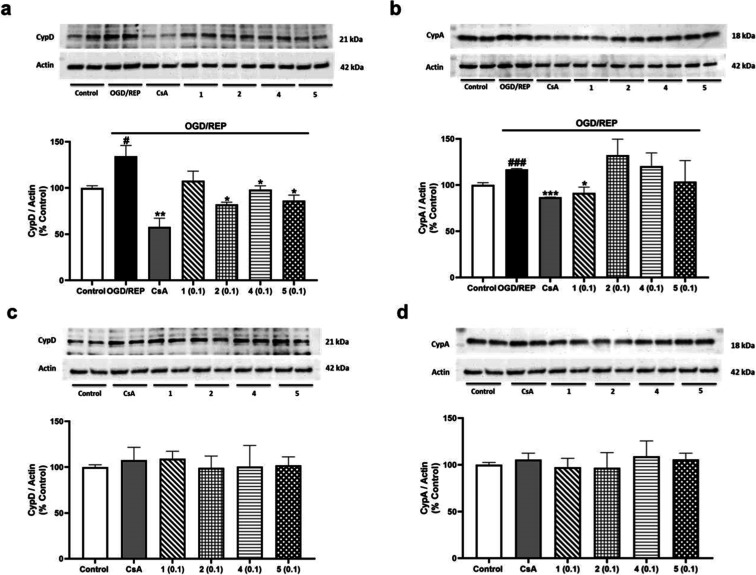
CypD and CypA expression after treatment with compounds **1**–**5** under OGD/REP conditions. Cells were pretreated with compounds **1**–**5** at 0.1 μM for 1 h and then incubated under OGD/REP. CsA (1 μM) was used as the positive control of Cyps route. Expression of CypD (a) and CypA (b) after the addition of compounds under OGD/REP. CypD (c) and CypA (d) intracellular expression in normoxia conditions after treatment with *S. tubulifera* metabolites. Band intensity was normalized by actin. Data are mean ± SEM of three independent experiments performed in triplicate. Data are expressed as percentage of normoxia control cells. Statistical differences were determined by one-way ANOVA and Dunnett’s tests. #*p* < 0.05 and ###*p* < 0.001 compared to normoxia control cells. *p < 0.05, **p < 0.01, and ***p < 0.001 compared to OGD/REP control cells.

Given the previous results, the effect of compounds **2**, **4**, and **5** in a coculture system that represents the cerebral microenvironment was analyzed. Thus, a *trans*-well coculture system was established with BV2 cells together with SH-SY5Y neuronal cells under OGD/REP conditions. The effect of compounds **2**, **4**, and **5** on the viability of neuroblastoma cocultured with activated microglia by OGD/REP was analyzed. The activation of microglia by the OGD/REP incubation produced a significant reduction of 30% in the viability of SH-SY5Y cells compared to cocultured neuroblastoma cells under normoxia conditions (Figure 9a). However, when SH-SY5Y cells were incubated under the OGD/REP alone, no effect over cell viability was observed (Figure 9b). In the coculture system under OGD/REP, pretreatment with compounds **2**, **4**, and **5** protected SH-SY5Y cells at 0.1 μM, reaching percentages between 106.4 and 120.7% of control cells. Treatment with CsA also protected neuroblastoma cells from activated microglia in these conditions (101.7 ± 2.0%) (Figure 9a).

**Figure 9:**
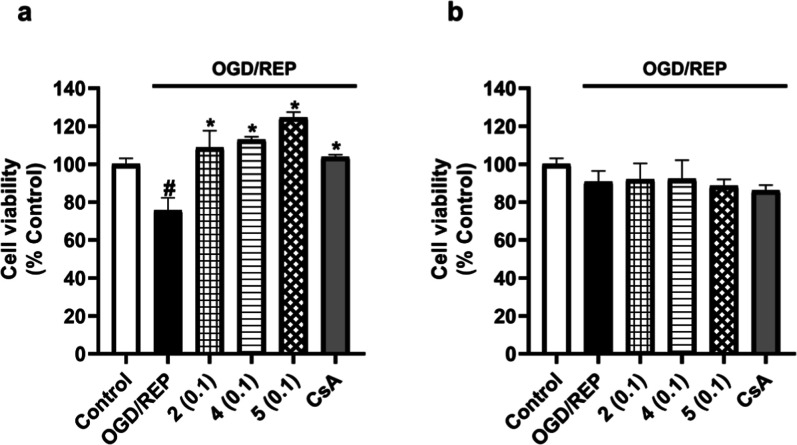
Effect of compounds **1**–**5** on the viability of SH-SY5Y cells in a microglia-neuron coculture system under OGD/REP incubation. SH-SY5Y and BV2 cells were cocultured and treated with compounds (0.1 μM) for 1 h and then incubated under OGD/REP. CsA (1 μM) was used as the positive control of Cyps route. Results of (a) SH-SY5Y cell viability cocultured with BV2 cells under OGD/REP conditions and treated with compounds **2**, **4**, and **5**. Results of (b) SH-SY5Y cell viability alone under OGD/REP conditions and treated with compounds **2**, **4**, and **5**. Data are mean ± SEM of three independent experiments performed in triplicate. Data are expressed as percentage of control cells. Statistical differences were determined by one-way ANOVA and Dunnett’s tests. #*p* < 0.05 compared to control cells. *p < 0.05 compared OGD/REP control cells.

## 4 Discussion and Conclusions

In this work, the effects of compounds **1**–**5** from *S. tubulifera* to protect BV2 microglial cells under ischemia/reperfusion conditions were described. Compounds showed antioxidant and mitochondrial-mediated protection against ischemic injury, which seems to be related to CypD inhibition. Currently, the only Cyps-modulating drug approved for clinical use is CsA, which has shown neurotoxicity, nephrotoxicity, and hepatotoxicity.^7^ In this way, compounds **1**–**5** can be considered potential candidates for ischemia-related diseases, which currently have no effective approved treatment.

Incubation under hypoxia reduced microglial viability after 6, 12, or 24 h in a time-dependent way, as previously described.^19^ Surprisingly, addition of the inflammatory stimulus LPS did not enhance the hypoxia-related injury. A previous study showed that LPS induces the endogenous expression of the anti-inflammatory mediator IL-10 in microglial cells, which could explain why LPS treatment in hypoxia conditions did not enhance the damage.^20^ In contrast, when microglia was under OGD and/or followed by REP, reduction in microglial viability was even higher than only under oxygen restriction, which is due to the increase of ischemic stress.^15^ Importantly, compounds **1**–**5** showed protective effects against hypoxia, OGD, or OGD/REP injury by mitigating the reduction of cell viability. Also, *S. tubulifera* furanoditerpenes neutralized the effect of hypoxia combined with inflammation after 6, 12, or 24 h. However, the control of CsA only showed an effect under OGD or OGD/REP conditions; therefore, this drug seems to be less effective under oxygen deprivation.

The protective effect of compounds **1**–**5** could be due to their ability to bind CypD, since the inhibition of this protein in hypoxic conditions reduces microglial activation and alleviates mitochondrial dysfunction.^3,21^ In our model of ischemia/reperfusion injury, we observed a significant increase in CypD expression, consistent with its role in promoting mPTP opening and in contributing to mitochondrial dysfunction.^22^ In these conditions, compounds **2**, **4**, and **5**, which are the ones with a higher affinity for CypD, significantly reduced CypD expression.^3^ These metabolites also induced a better recovery of mitochondrial function, as evidenced by the reduction in ROS levels and the preservation of ΔΨm, which is compromised in hypoxia and OGD/REP conditions.^15,23^ Consistently, compounds **2**, **4**, and **5** were also the most effective ones reducing ROS levels and mitochondrial depolarization in neuronal cells subjected to oxidative stress.^3^ In contrast, as evidenced in our results, compounds **1** and **3** showed better outcomes under oxygen deprivation than after reperfusion. These differences between compounds **1**–**5** reflect variances in CypD modulation, as described before. According to this, previous findings reported that compounds **2** and **5** had higher CypD affinity and compounds **4** and **5** inhibited mPTP opening.^3^ Moreover, these metabolites increased the glutathione content in neurons, an antioxidant system that counteracts ROS production and is decreased in microglia exposed to hypoxia.^3,24^ Taken together, these results support the idea that the protective properties of *S. tubulifera* metabolites in ischemia are mediated by CypD inhibition.

In a coculture system with SH-SY5Y neuronal cells, compounds **2**, **4**, and **5** attenuated the reduction in cellular viability induced by BV2 microglia under the OGD/REP conditions. Microglial activation triggers the release of pro-inflammatory cytokines and neurotoxic mediators which contribute to neuronal injury.^25^ Therefore, the improvement in neuronal viability could be due to the inhibition of microglial activation by *S. tubulifera* metabolites through CypD inhibition. This protein is also related to inflammation since CypD deficiency decreases the inflammatory response.^26^

The modulation of CypD produced by compounds could be related to their chemical structures.^3^ Our results suggest that the presence of a hydroxyl group at C-19 in compounds **4** and **5** is critical for counteracting reperfusion-associated damage. Moreover, the presence of a Δ^1^ double bond along with a hydroxyl group at C-2 and a ketone carbonyl functionality at C-3 in compounds **2** and **5** could also be responsible for the protective effect of these compounds under OGD/REP.

Finally, compound **1** showed no effect in CypD levels, which could explain why the response of this compound is lower after reperfusion.^27^ In contrast, compound **1** was the only metabolite from *S. tubulifera* that reduced the increase in CypA expression, agreeing with its selectivity for CypA binding.^3^ In this sense, compound **1** was also the only metabolite that reduced CypA expression in neuronal cells exposed to oxidative stress.^3^ In previous studies, CypA upregulation has been related to ROS production, so the ability of compound **1** to reduce ROS levels and thereby in preserving mitochondrial function could be due to its selective affinity for CypA.^3,28^

In conclusion, compounds **1**–**5** from *S. tubulifera* exhibited mitochondrial-mediated protective effects against ischemia through inhibition of CypD. In the OGD/REP conditions, which mimic the pathophysiology of an ischemia event, compounds **2**, **4**, and **5** were the most promising ones, recovering mitochondrial function and reducing ROS levels. Finally, the attenuation of neuronal viability loss in the coculture supports the potential of compounds **1**–**5** as promising therapeutic candidates for the prevention of ischemic injury, either in stroke or during organ transplantation.

## Data Availability

Data will be made available on request.

## Abbreviations

ATCC: American Type Culture Collection
carboxy-H_2_DCFDA: 5-(and 6)-carboxy-2′,7′-dichlorodihydrofluorescein diacetate
CNS: central nervous system
CsA: cyclosporine A
Cyp: cyclophilin
DMEM/F-12: Dulbecco’s modified Eagle’s medium: Nutrient Mix F-12
FBS: fetal bovine serum
ICLC: interlab cell line collection
LPS: lipopolysaccharide
mPTP: mitochondrial permeability transition pore
MTT: 3-(4,5-dimethylthiazol-2-yl)-2,5-diphenyl tetrazolium bromide
OGD: oxygen glucose deprivation
OGD/REP: oxygen glucose deprivation/reperfusion
PBS: phosphate-buffered saline
ROS: reactive oxygen species
SDS: sodium dodecyl sulfate
TMRM: tetramethylrhodamine methyl ester
ΔΨm: mitochondrial membrane potential
